# Biotic and Paleoceanographic Changes Across the Late Cretaceous Oceanic Anoxic Event 2 in the Southern High Latitudes (IODP Sites U1513 and U1516, SE Indian Ocean)

**DOI:** 10.1029/2022PA004474

**Published:** 2022-09-08

**Authors:** Maria Rose Petrizzo, Giulia Amaglio, David K. Watkins, Kenneth G. MacLeod, Brian T. Huber, Takashi Hasegawa, Erik Wolfgring

**Affiliations:** ^1^ Dipartimento di Scienze della Terra “A. Desio” Università degli Studi di Milano Milano Italy; ^2^ Department of Earth and Atmospheric Sciences University of Nebraska Lincoln NE USA; ^3^ Department of Geological Sciences University of Missouri‐Columbia Columbia MO USA; ^4^ National Museum of Natural History Smithsonian Institution Washington DC USA; ^5^ Faculty of Geosciences and Civil Engineering Institute of Science and Engineering Kanazawa University Kanazawa Japan; ^6^ Department of Geology University of Vienna Vienna Austria

**Keywords:** Oceanic Anoxic Event 2, stable isotopes, paleoceanography, planktonic foraminifera, calcareous nannofossils, radiolaria, calcispheres, benthic foraminifera

## Abstract

Oceanic Anoxic Event 2, spanning the Cenomanian/Turonian boundary (93.9 Ma), was an episode of major perturbations in the global carbon cycle. To investigate the response of biota and the paleoceanographic conditions across this event, we present data from International Ocean Discovery Program sites U1513 and U1516 in the Mentelle Basin (offshore SW Australia; paleolatitude 59°–60°S in the mid‐Cretaceous) that register the first complete records of OAE 2 at southern high latitudes. Calcareous nannofossils provide a reliable bio‐chronostratigraphic framework. The distribution and abundance patterns of planktonic and benthic foraminifera, radiolaria, and calcispheres permit interpretation of the dynamics of the water mass stratification and provide support for the paleobathymetric reconstruction of the two sites, with Site U1513 located northwest of the Mentelle Basin depocenter and at a deeper depth than Site U1516. The lower OAE 2 interval is characterized by reduced water mass stratification with alternating episodes of enhanced surface water productivity and variations of the thickness of the mixed layer as indicated by the fluctuations in abundance of the intermediate dwelling planktonic foraminifera. The middle OAE 2 interval contains lithologies composed almost entirely of radiolaria reflecting extremely high marine productivity; the low CaCO_3_ content is consistent with marked shoaling of the Carbonate Compensation Depth and ocean acidification because of CaCO_3_ undersaturation. Conditions moderated after deposition of the silica‐rich, CaCO_3_‐poor rocks as reflected by the microfossil changes indicating a relatively stable water column although episodes of enhanced eutrophy did continue into the lower Turonian at Site U1516.

## Key Points

- Documentation of first complete record of the Late Cretaceous Oceanic Anoxic Event 2 (OAE 2) at southern high latitudes (60°S) in the Indian Ocean
- Dynamics of the water mass stratification inferred from distribution patterns of foraminifera, radiolaria, calcispheres
- OAE 2 is characterized by alternating episodes of enhanced surface water productivity and variations of the thickness of the mixed layer

## 1 Introduction

The latest Cenomanian‐earliest Turonian Oceanic Anoxic Event 2 (e.g., Jenkyns et al., 2017) represents the last truly global oceanic anoxic event and approximately coincides with the maximum global warmth of the Late Cretaceous (e.g., Forster et al., 2007; Friedrich et al., 2012; Huber et al., 2018; O’Brien et al., 2017; Wilson et al., 2002). The classic lithologic expression of OAE 2 is a shift to deposition of organic‐rich shales and marls in hemipelagic and pelagic settings nearly worldwide (e.g., Scholle & Arthur, 1980; Schlanger & Jenkyns, 1976; Schlanger et al., 1987), whereas its primary geochemical signature is a synchronous positive δ^13^C excursion in both carbonates and organic matter that is also identifiable worldwide (e.g., Jenkyns, 2010; Jenkyns et al., 2017; Robinson et al., 2017; Tsikos et al., 2004; Voigt et al., 2006; Wendler, 2013). This isotopic excursion results from the net burial of large amounts of organic matter in deep‐sea and hemipelagic settings (e.g., Jenkyns, 2010; Jenkyns et al., 2017).

Causes for OAE 2 are still the subject of investigations; however, several studies postulate that massive submarine volcanic activity (i.e., emplacement of the Caribbean, High Arctic, and/or the Kerguelen Plateau Large Igneous Province) emitted greenhouse gases and provided biolimiting metals in marine ecosystems leading to the onset of the Cenomanian‐Turonian Thermal Maximum and the enhancement of ocean fertility (e.g., Barclay et al., 2010; Du Vivier et al., 2014; Erba, 2004; Gangl et al., 2019; Jenkyns, 2003; Jiang et al., 2021; Kuroda et al., 2007; Kuypers et al., 2002; Larson, 1991; Leckie et al., 2002; Matsumoto et al., 2022; Pancost et al., 2004; Scaife et al., 2017; Schröder‐Adams et al., 2019; Trabucho Alexandre et al., 2010; Turgeon & Creaser, 2008; Zheng et al., 2013, among many others). Ocean temperature, sea‐surface stratification, nutrient availability, carbonate ion saturation and continental weathering were also subject to significant variations during OAE 2 (Jenkyns, 2010, and references therein), and these changes certainly influenced the geographic distribution and abundance of marine species according to their paleoecological preferences.

Planktonic foraminifera across the Cenomanian‐Turonian boundary interval studied in several low to mid latitude regions of the Tethyan Realm (e.g., England: Falzoni & Petrizzo, 2020; Keller et al., 2001; Paul et al., 1999; Austria: Gebhardt et al., 2010; Wagreich et al., 2008; Italy: Coccioni & Luciani, 2004, 2005; Luciani & Cobianchi, 1999; Scopelliti et al., 2004, 2008; SE France: Falzoni & Petrizzo, 2022; Falzoni, Petrizzo, Jenkyns, et al., 2016; Grosheny et al., 2006; Takashima et al., 2009; Spain: Lamolda et al., 1997; Tunisia and Algeria: Benadla et al., 2018; Caron et al., 2006; Grosheny et al., 2013; Nederbragt & Fiorentino, 1999; Reolid et al., 2015; Zaghbib‐Turki & Soua, 2013; Iran: Kalanat et al., 2016; Kalanat & Vaziri‐Moghaddam, 2019; Morocco: Aquit et al., 2013; Falzoni et al., 2018; Keller et al., 2008), of the central Atlantic Ocean (Nigeria: Gebhardt, 1997; Morocco: Jati et al., 2010; Mexico: Ifrim et al., 2011), of the Indian Ocean (Tibet: Bomou et al., 2013), of the Pacific Ocean (Japan: Hasegawa, 1999), and of the Western Interior Seaway (e.g., USA: Caron et al., 2006; Desmares et al., 2007; Elderbak & Leckie, 2016; Elderbak et al., 2014; Keller & Pardo, 2004; Canada: Dionne et al., 2016; Bryant et al., 2021) underwent a significant change.

Notable events are the extinction of the deeper dwelling, single‐keeled rotaliporids (including *Thalmanninella deeckei*, *Thalmanninella greenhornensis*), whose last representative *Rotalipora cushmani* disappeared shortly after the onset of OAE 2 (e.g., Falzoni et al., 2018; Leckie et al., 2002; Premoli Silva & Sliter, 1999) likely because of warming of deep waters leading to reduced vertical stratification (Huber et al., 1999) and the evolution and diversification of the mixed layer to thermocline dwelling, double‐keeled *Dicarinella* and *Marginotruncana* that dominated the assemblages from the time of OAE 2 until the Santonian (e.g., Falzoni, Petrizzo, Clarke, et al., 2016; Petrizzo, 2000, 2002; Petrizzo et al., 2017; Premoli Silva & Sliter, 1995, 1999). Another remarkable event is the abrupt increase in abundance of the opportunistic biserial planktonic foraminifera (i.e., *Planoheterohelix*) associated with the peak OAE 2. This event, known as *Heterohelix* shift (Leckie, 1985; Leckie et al., 1998), is documented from many localities in the low to middle latitude records (e.g., England: Keller et al., 2001; Morocco: Falzoni et al., 2018; Keller et al., 2008; Umbria‐Marche Basin, Italy: Coccioni & Luciani, 2004, 2005; Tunisia: Caron et al., 2006; Nederbragt & Fiorentino, 1999; Zagrarni et al., 2008; Iran: Kalanat & Vaziri‐Moghaddam, 2019; Japan: Hasegawa, 1997; Hasegawa et al., 2013; Western Interior Seaway: Bryant et al., 2021; Elderbak & Leckie, 2016; Leckie, 1985; Leckie et al., 1998, among many others).

In general, within the OAE 2 interval planktonic foraminiferal assemblages are low in diversity and indicative of increased sea‐surface productivity and are often absent or very rare in the organic‐rich layers deposited during OAE 2 (e.g., Caron et al., 2006; Coccioni & Luciani, 2004, 2005; Coccioni et al., 2006; Falzoni, Petrizzo, Jenkyns, et al., 2016; Grosheny et al., 2006, 2013; Keller & Pardo, 2004; Keller et al., 2001, 2008; Kopaevich & Vishnevskaya, 2016; Leary et al., 1989; Leckie, 1985, 1987; Leckie et al., 1998, 2002; Luciani & Cobianchi, 1999; Nederbragt & Fiorentino, 1999; Paul et al., 1999; Petrizzo et al., 2021; Premoli Silva et al., 1999; Reolid et al., 2016; Scopelliti et al., 2004, 2008, among many others).

At high latitudes in the Southern Hemisphere, changes across OAE 2 are not well known. The sedimentary record of the region is often incomplete because of hiatuses (Kerguelen Plateau: Dickson et al., 2017) or poor recovery of sediments (Naturaliste Plateau: Luyendyk & Davies, 1974), and planktonic foraminifera are reported to be either rare and characterized by low diversity (Exmouth Plateau: Wonders, 1992; Kerguelen Plateau: Petrizzo, 2001; Naturaliste Plateau: Herb, 1974; Huber et al., 2018) or are absent (New Zealand: Hasegawa et al., 2013). Planktonic foraminifera occur across the Cenomanian‐Turonian boundary interval in the Cauvery and Narmada basins in India (Keller et al., 2021; Tewari et al., 1996), but they have not been studied in detail relative to the OAE 2.

**Figure 1:**
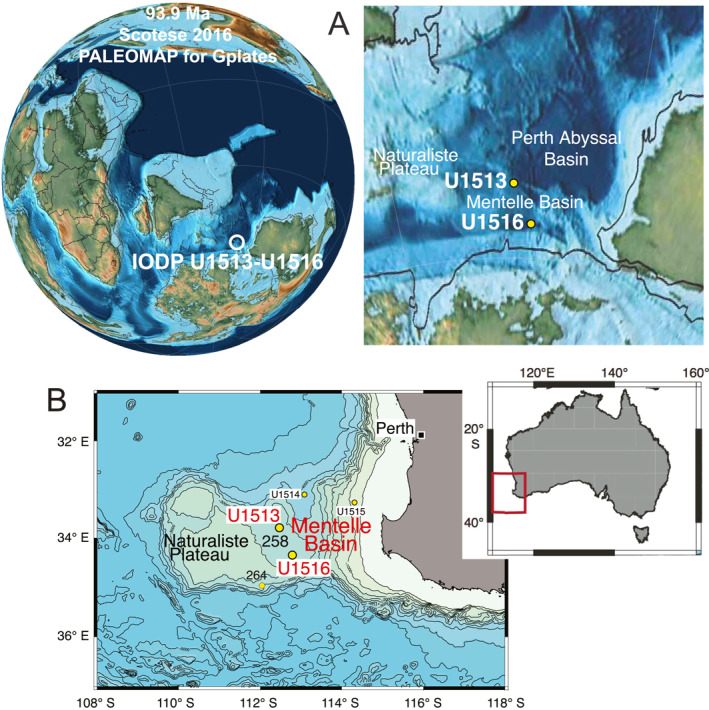
(A) Paleogeographic map of the late Cenomanian (93.9 Ma) with positions of International Ocean Discovery Program (IODP) Expedition 369 sites U1513 and U1516 (Scotese, 2016). (B) Locations of the sites drilled during IODP Expedition 369 and Deep Sea Drilling Project Leg 26 sites 258 and 264 (modified after Huber et al., 2019a).

Previous study of a complete Cenomanian‐Turonian boundary interval during International Ocean Discovery Program (IODP) Expedition 369 at Site U1516 in the Mentelle Basin (Figure 1) has generated the best high latitude record of OAE 2 (Huber et al., 2019a), allowing reconstruction of a robust bio‐chemostratigraphic framework for interpreting the paleoceanographic fluctuations based on changes in the planktonic foraminiferal assemblages and the co‐occurring benthic foraminifera and radiolaria (Petrizzo et al., 2021).

The Mentelle Basin, located off southwest Australia on the eastern flank of the Naturaliste Plateau (Figure 1), is a rifted continental margin basin that accumulated sediments eroded from surrounding continents as well as volcanogenic sediments sourced from the junction between Australia, Antarctica and Greater India during the final stage of the Gondwana breakup (Borissova et al., 2010). It preserves sediments deposited in a thermally subsiding basin after the initial breakup of East Gondwana in the late Valanginian (Harry et al., 2020; Huber et al., 2019a; Lee et al., 2020; Tejada et al., 2020; Wainman et al., 2019).

This study is focused on planktonic foraminiferal population dynamics, the benthic foraminifera, radiolaria and calcispheres distributions and the carbon isotope record across the Cenomanian‐Turonian boundary interval and OAE 2 at Site U1513 and their comparison of these observations with results from Site U1516 previously published in Petrizzo et al. (2021). Considered together, data from the two sites, located 69 km apart in the Mentelle Basin (Figure 1), test the synchroneity of details of the response of the microbiota to the paleoenvironmental perturbation associated with the δ^13^C excursion and highlight similarities and differences between the sites.

## 2 Materials and Methods

IODP Sites U1513 (33°47.6084′S, 112°29.1338′E) and U1516 (34°20.9169′S, 112°47.9553′E) lie at 2,800 and 2,676.6 m water depth, respectively, on the western margin of the Mentelle Basin and off the southwestern margin of Australia in the southeast Indian Ocean (Huber et al., 2019a). The sites were situated at about 59°–60°S latitude during the Late Cretaceous (Hay et al., 1999; Muller et al., 2016; Scotese, 2016; van Hinsbergen et al., 2015) (Figure 1).

This study focuses on the upper Cenomanian to lowermost Turonian sedimentary sequence recovered from holes U1513A (core 39X to core 48X), U1513D (core 15R to core 22R) and from holes U1516C (core 28R to core 35R) and U1516D (core 2R to core 5R) previously studied by Petrizzo et al. (2021). The overlapping portions of sites U1513 and U1516 together provide relatively continuous recovery of the Cenomanian‐Turonian boundary interval. The two sites were drilled during IODP Expedition 369 in 2017 on the eastern flank of the Naturaliste Plateau and are 69 km apart. Moreover, Site U1513 is located 1.1 km east‐northeast of Deep Sea Drilling Project (DSDP) Leg 26 Site 258 (Figure 1), where the Cretaceous interval was spot‐cored. Coring at Site 258 recovered an incomplete OAE 2 interval (Huber et al., 2018; Luyendyk and Davies, 1974). All Site U1516 information and data reported in this study are from Petrizzo et al. (2021) except when otherwise stated.

**Figure 2:**
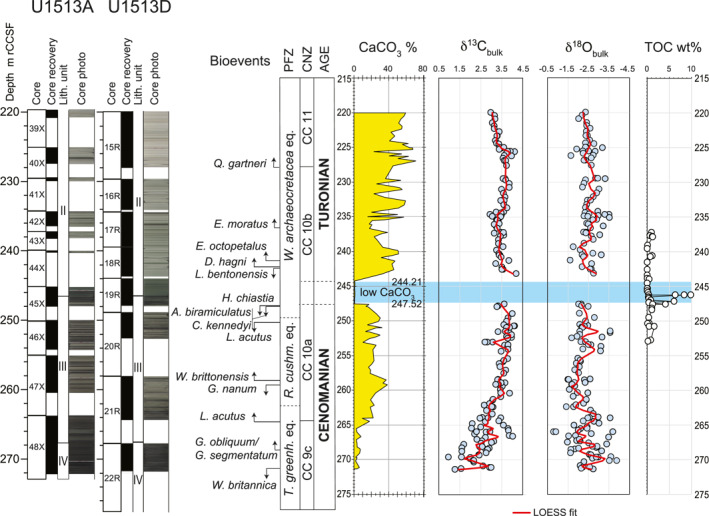
Holes U1513A and U1513D. Core recovery, lithologic units, core photos according to Huber et al. (2019a). Calcareous nannofossils and planktonic foraminifera biostratigraphy, age, CaCO_3_ content, δ^13^C and δ^18^O, and Total Organic Carbon (TOC) according to this study. LOESS fit: see explanation in the text. Abbreviations: m rCCSF = revised Core Composite depth below Sea Floor in meters; PFZ = Planktonic Foraminifera Zones; CNZ = Calcareous Nannofossils Zones; *W*. *archaeocretacea* eq. = *Whiteinella archaeocretacea* equivalent; *R*. *cushm*. eq. = *Rotalipora cushmani* equivalent; *T*. *greenh*. eq. = *Thalmanninella greenhornensis* equivalent.

Data are plotted in meters on the rCCSF depth scale (revised Core Composite depth below Sea Floor, equivalent to mcd meters composite depth) revised by S. Batenburg (pers. comm. 2020) from the shipboard splice (Huber et al., 2019a, 2019b; LIMS online report portal at http://web.iodp.tamu.edu/LORE/). The lithologies in Hole U1513A (core 39X to core 45X‐2) and in Hole U1513D (core 15R to core 19R‐2) are assigned to lithostratigraphic Unit II (Figure 2) that is composed of alternating sequences of medium to thick, sparsely to intensely bioturbated beds of green, gray and black nannofossil‐rich claystone. The underlying lithostratigraphic Unit III from core 45X‐2, 10 cm to core 48X‐3, 35 cm in Hole U1513A and from core 19R‐2, 96 cm to core 21R‐CC in Hole U1513D consist of alternating sequences of greenish gray, very dark greenish gray and black claystone. Sediments from core 48X‐3, 35 cm in Hole U1513A and core 22R in Hole U1513D are assigned to lithostratigraphic Unit IV which is composed of a sequence of massive to mottled dark greenish gray and black nannofossils claystone. Lithologies at Site U1516 are assigned to lithostratigraphic Unit II, III and IV that are equivalent to those described at Site U1513 as explained in Huber et al. (2019a) and Petrizzo et al. (2021).

For documenting the stratigraphic distribution and absolute abundances of microfossil groups as well as the planktonic foraminiferal species richness, 111 rock samples of about 10 cm^3^ from Site U1513 were processed at the University of Milan and studied according to the procedure and methodology explained in Petrizzo et al. (2021) and Petrizzo, MacLeod et al. (2022). Specifically, rock samples were dried, weighed and soaked in a solution of water and H_2_O_2_, washed over 250, 125, and 38 μm sieves and dried to obtain washed residues. For each size fraction microfossil groups were counted in statistically reliable splits of the washed residues. Planktonic foraminifera species richness was calculated for the >38 μm size fraction. The number of specimens for each category was multiplied by the number of splits and the total number of specimens were obtained for each sample by adding the values of the three size fractions. Absolute abundances of planktonic and benthic foraminifera and of radiolaria and calcispheres were calculated as the number of specimens per gram of dry sediment. Microfossils counts for Site U1513 are reported in Petrizzo, Amaglio, et al. (2022). For Site U1516 microfossils counts are from Petrizzo et al. (2021). Planktonic foraminifera taxonomy follows the pforams@mikrotax database at http://www.mikrotax.org/pforams (Huber et al., 2016) and Huber et al. (2022). The planktonic foraminiferal biozonation follows Robaszynski and Caron (1995) and Petrizzo and Gilardoni (2020). The benthic foraminiferal taxonomy follows Loeblich and Tappan (1988), Belford (1959, 1960), Quilty (1992), Widmark and Speijer (1997), and Kaiho (1998). The most common taxa were photographed using Scanning Electron Microscopy (SEM Jeol JSM‐IT500) at the University of Milan.

Eighty two nannofossil‐bearing samples from U1513A (core 40X to 48X) and U1513D (core 15R –22R) spanning approximately 46.7 m of the composite section were prepared at the University of Nebraska and studied for biostratigraphic determination. At least 400 fields of view at 1,000x were observed using light microscopy for each sample to calculate species richness and identify the distributions of biostratigraphically‐significant taxa. The biozonation for calcareous nannofossils is according to Perch‐Nielsen (1985). Ages were assigned to biostratigraphic datums following Gradstein et al. (2012). Calcareous nannofossils data are reported in Petrizzo, Amaglio, et al. (2022).

The 111 samples from Site U1513 used to quantify foraminiferal, radiolarian and calcispheres populations were also measured for CaCO_3_ content. Values were chemically detected using a Dietrich–Frühling calcimeter at the University of Milan that measures the volume of CO_2_ developed by hydrochloric acid reacting with the bulk sample, which is proportional to the carbonate concentration. CaCO_3_ results are reported in weight percentage (%) and included in Petrizzo, Amaglio, et al. (2022).

Stable isotope values for bulk carbonate from Site U1513 were measured at the University of Milan and at the University of Missouri. Carbon and oxygen isotope analyses of carbonate powder subsampled from the 111 samples above were measured at the University of Milan using an automated carbonate preparation device (GasBench II) connected to a Delta V Advantage (Thermo Fisher Scientific Inc.) isotopic ratio mass spectrometer. Carbonate powders (about 200 μg each) were reacted with >99% orthophosphoric acid at 70°C for 1 hr. The carbon and oxygen isotope compositions are expressed in the conventional delta notation calibrated to the Vienna Pee‐Dee Belemnite scale by the international standards NBS18 and IAEA‐603 as well as internal standards (Carrara marble) during each run of samples. Analytical reproducibility for these analyses was better than ±0.1‰ for both δ^18^O and δ^13^C values. An additional 59 samples bulk carbonate samples were measured at the University of Missouri using a Kiel III carbonate device connected to a DeltaPlus isotope ratio mass spectrometer. Method details and data are presented in Edgar et al. (2022). Analytical reproducibility for these analyses was better than ±0.03‰ for δ^13^C values and 0.06‰ for δ^18^O values. The bulk carbonate isotope data are included in Petrizzo, Amaglio, et al. (2022).

Total Organic Carbon (TOC) content was measured in 40 samples at Kanazawa University. About 40 mg of each sample was powdered using a mortar and pestle and reacted with 1 M HCl for 8 hours to remove carbonate, then washed with deionized water to neutralize, and dried. Approximately 2 mg of the powder was placed in a tin film cup and weighed. The samples were combusted at about 1,000°C in a Thermoquest NA2500 elemental analyzer, and the produced CO_2_ was detected with TCD. For calibration, 2.5‐bis (5‐tert‐butylbenzoxazol‐2‐yl) thiophene (BBOT) was used as a standard. A K‐factor method was employed to obtain a regression line for quantitative analyses. Each TOC value was expressed as a percentage of dry total sample weight, and the value was calculated as the mean of three analyses on a sample. The TOC data are included in Petrizzo, Amaglio, et al. (2022).

## 3 Results: Site U1513

### 3.1 CaCO_3_ Content and Isotope Data From Bulk Carbonate and TOC

The carbonate content in the studied sequence decreases dramatically in the interval from 244.21 to 247.52 m rCCSF. This interval consists of black nannofossil‐rich claystone and yield very low CaCO_3_ values ranging from 0.2% to 0.6% (Figure 2; Petrizzo, Amaglio, et al., 2022). Low CaCO_3_ contents (average 5%) are also observed at the base of the stratigraphic section from 265.52 to 271.31 m rCCSF which coincides with a lithologic shift to massive black nannofossil claystone. In the overlying interval the CaCO_3_ content increases to about 30% before the abrupt drop to 0.4% at 247.52 m rCCSF. The interval above 244.21 m rCCSF is composed of an alternating sequence of gray and black nannofossil‐rich claystone. In this upper interval CaCO_3_ content increases with values fluctuating from 15% to 71% (Figure 2; Petrizzo, Amaglio, et al., 2022).

Stable isotope values for bulk carbonate (178 analyses from 170 samples; Figure 2; Petrizzo, Amaglio, et al., 2022) show generally good reproducibility between laboratories and little difference in average values above and below the interval of low CaCO_3_ content (Figure 2). The lower part of the studied interval from 262.15 to 271.41 m rCCSF shows high variability in δ^13^C and δ^18^O with values ranging from 0.95‰ to 4.02‰ and from −0.7‰ to −3.8‰, respectively. The high variability and low values observed in this interval may reflect a significant contribution from benthic bioclasts and the presence of diagenetic carbonate. The δ^13^C values through the rest of the section fluctuate by 0.5‰. The positive δ^13^C excursion recorded at Site U1516 (Petrizzo et al., 2021), which correlates with the low CaCO_3_ content interval, is not observed at Site U1513. Within the same interval in the rest of the section δ^18^O values fluctuate by 1.0‰ and range from −1.5‰ to −3.5‰ without showing considerable positive or negative variations (Figure 2). To highlight trends in the bulk carbonate data, trendlines were calculated using the LOESS function in Matlab based on a series of quadratic polynomial ﬁts to each point and its 10 closest neighbors weighted by proximity to the point being evaluated (Figure 2; Petrizzo, Amaglio, et al., 2022).

The TOC content (Figure 2; Petrizzo, Amaglio, et al., 2022) shows peak values ranging from 6.34 wt% to 9.91 wt% within the low CaCO_3_ content interval from 246.26 to 246.30 m rCCSF. Minor peaks of high values of 6.15 wt% and 4.17 wt% are registered at 247.19 and 247.60 m rCCSF, respectively. In the rest of the studied stratigraphic interval TOC values range from 0.07 wt% to 0.89 wt% (Figure 2).

### 3.2 Bio‐Chronostratigraphic Framework, Age‐Depth Model and Sedimentation Rates

The calcareous plankton assemblages at Site U1513 show similar features to those documented at nearby Site U1516 (Petrizzo et al., 2021) and are characterized by the absence of many typical mid‐ to low latitude and age diagnostic taxa useful for constraining the Cenomanian/Turonian boundary. Nevertheless, a reliable bio‐chronostratigraphic framework based on calcareous nannofossils has been obtained.

**Figure 3:**
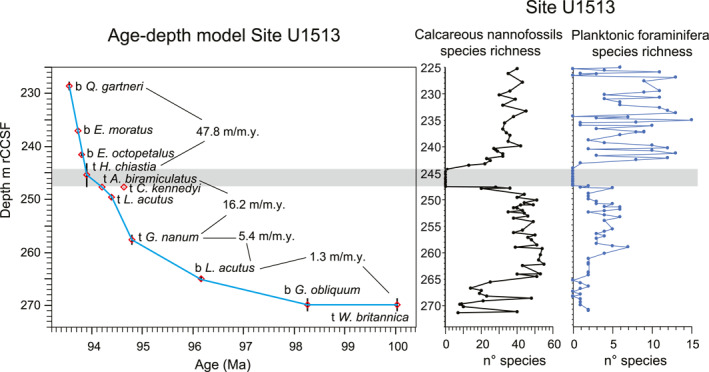
Age‐depth model for Site U1513 build using calcareous nannofossil bioevent ages (Gradstein et al., 2012). Calculation of the best‐fit line of correlation drawn through the bioevents that are deemed to be most reliable provide a means for calculating the sedimentation rates (m/m.y.). Stratigraphic uncertainties are plotted as vertical bar for each datum. Calcareous nannofossils and planktonic foraminifera species richness are shown, see explanation in the text. Gray band = interval of low CaCO_3_ content barren of calcareous nannofossils and planktonic foraminifera. Abbreviations: t = top; b = base.

The base of the studied stratigraphic interval from 264.47 to 271.41 m rCCSF (Cores U1513A‐48X and U1513D‐22R; Figure 2) consists largely of calcareous mudstones with variable, but generally low carbonate content that range from maximum value of 17% at the top of the interval to a minimum value of 1.3% near the base (Figure 2; Petrizzo, Amaglio, et al., 2022). Many samples contain high abundances of phillipsite crystals (euhedral to subhedral), indicating substantial silica diagenesis consistent with the generally moderate to poor preservation of calcareous plankton and dominance of radiolaria in these samples. Planktonic foraminifera are rare, are present in low diversity (1‐2 species, Figure 3; Petrizzo, Amaglio, et al., 2022) and are moderately preserved. Age diagnostic species are absent. Calcareous nannofossil species richness in this interval is often low (<20 species, Figure 3; Petrizzo, Amaglio, et al., 2022), and the assemblage are dominated by dissolution‐resistant taxa.

Biostratigraphic control for this interval is based largely on three samples which exhibit higher calcareous nannofossil richness and significantly better preservation than the others. The lowest good sample (U1513D‐22R‐3, 25–28 cm; 271.13 m rCCSF; Figure 2) contains both *Watznaueria britannica* and *Corollithion kennedyi*, the co‐occurrence of which corresponds to a short interval in the earliest Cenomanian from 100.0 to 100.5 Ma (Table 1) assigned to the nannofossil Subzone CC 9c (Figure 2). The next highest sample containing moderately well‐preserved microfossils occurs at 268.57 m rCCSF (U1513A‐48X‐4, 30–33 cm) and contains *C*. *kennedyi* as well as the first appearance datum (FAD) of *Gartnerago obliquum* and *Gartnerago segmentatum*, which is placed at 98.3 Ma (Figure 2, Table 1). The third sample providing good biostratigraphic control is U1513A‐48X‐1, 65–68 cm at 264.47 m rCCSF (Figure 2). It occurs at the top of the mudstone interval and contains the FAD of *Lithraphidites acutus* (96.2 Ma; Table 1). The FAD of this species has been widely used in Cenomanian biostratigraphy to identify the base of Subzone CC 10a; however, *L*. *acutus* is extremely rare at Site U1513. It is present in only about 25% of the samples examined within its apparent stratigraphic range and, where present, was only identified by single specimens. Accumulation rates for this interval are approximately 1.3 m/m.y. as indicated by the age‐depth plot (Figure 3).

**Table 1:** Calcareous Plankton Datum and Ages at Sites U1513 and U1516

SITE U1513		TOP	TOP m rCCSF	BOTTOM	BOTTOM m rCCSF	MEAN DEPTH m rCCSF	AGE
Event		Sample		Sample			
FAD	*Quadrum gartneri*	U1513D‐15R‐CC, 7–12 cm	227.80	U1513C‐41X‐CC, 27–30 cm	229.47	228.64	93.55
FAD	*Eprolithus moratus*	U1513D‐17R‐2, 120–123 cm	236.65	U1513C‐43X‐1, 35–38 cm	237.35	237.00	93.73
FAD	*Eprolithus octopetalus*	U1513D‐18R‐2, 95–98 cm	241.33	U1513D‐18R‐2, 140–143 cm	241.78	241.55	93.79
LO	*Dicarinella hagni*	U1513D‐18R‐3, 35–38 cm	242.23	U1513D‐18R‐3, 71–74 cm	242.59	242.41	nr
HO	*Laeviella bentonensis*	U1513D‐18R‐3, 35–38 cm	242.23	U1513D‐18R‐3, 71–74 cm	242.59	247.56	nr
LAD	*Helenea chiastia*	U1513D‐18R‐3, 133–136 cm	243.21	U1513C‐45X‐2, 144–147 cm	247.60	245.40	93.90
HO	*Axopodorhabdus biramiculatus*	U1513C‐45X‐2, 144–147 cm	247.60	U1513C‐45X‐3, 16–18 cm	247.81	247.71	94.20
HO	*Corollithion kennedyi*	U1513C‐45X‐2, 144–147 cm	247.60	U1513C‐45X‐3, 16–18 cm	247.81	247.71	nr
LAD	*Lithraphidites acutus*	U1513D‐20R‐1, 37–40 cm	248.97	U1513D‐20R‐1, 145–148 cm	250.05	249.51	94.40
LAD	*Gartnerago nanum/ponticula*	U1513C‐47X‐1, 140–143 cm	256.65	U1513C‐47X‐2, 135–138 cm	258.49	257.57	94.79
LO	*Whiteinella brittonensis*	U1513D‐21R‐1, 100–103 cm	259.20	U1513C‐47X‐3, 78–81 cm	260.38	259.79	nr
FAD	*Lithraphidites acutus*	U1513C‐48X‐1, 65–68 cm	264.47	U1513C‐48X‐2, 25–28 cm	265.52	265.00	96.16
FAD	*Gartnerago obliquum/segmentatum*	U1513C‐48X‐4, 30–33 cm	268.57	U1513D‐22R‐3, 25–28 cm	271.13	269.85	98.26
LAD	*Watznaueria britannica*	U1513C‐48X‐4, 30–33 cm	268.57	U1513D‐22R‐3, 25–28 cm	271.13	269.85	100.03
LO	*Corollithion kennedyi*	U1513D‐22R‐3, 25–28 cm	271.13	U1513C‐48X‐6, 65–68 cm	271.31	271.22	100.45

SITE U1516		TOP	TOP m rCCSF	BOTTOM	BOTTOM m rCCSF	MEAN DEPTH m rCCSF	AGE
Event		Sample		Sample			
FAD	*Quadrum gartneri*	U1516C‐30R‐2, 95–96 cm	461.68	U1516C‐30R‐2, 134–137 cm	462.06	461.87	93.55
FAD	*Eprolithus moratus*	U1516C‐30R‐3, 71–75 cm	462.92	U1516C‐30R‐3, 131–133 cm	463.48	463.20	93.73
LO	*Dicarinella hagni*	U1516D‐3R‐1, 121–123 cm	464.14	U1516D‐3R‐2, 15–18 cm	464.39	464.27	nr
FAD	*Eprolithus octopetalus*	U1516C‐31R‐2, 8–11 cm	465.79	U1516C‐31R‐2, 97–100 cm	466.68	466.24	93.79
LAD	*Helenea chiastia*	U1516C‐31R‐2, 8–11 cm	465.79	U1516C‐32R‐1, 97–100 cm	470.89	468.34	93.90
LO	*Whiteinella brittonensis*	U1516D‐4R‐2, 98–101 cm	469.26	U1516C‐31R‐4, 82–85 cm	469.87	469.56	nr
LAD	*Axopodorhabdus biramiculatus*	U1516C‐32R‐1, 131–134 cm	471.24	U1516C‐32R‐2, 4–7 cm	471.48	471.36	94.20
LAD	*Lithraphidites acutus*	U1516C‐32R‐3, 111–113 cm	474.05	U1516C‐32R‐CC, 1–5 cm	474.12	474.09	94.40
HO	*Laeviella bentonensis*	U1516C‐32R‐3, 111–113 cm	474.05	U1516C‐32R‐CC, 0–5 cm	474.11	474.08	nr
LAD	*Gartnerago nanum*	U1516C‐33R‐5, 3–7 cm	479.29	U1516C‐33R‐CC, 1–5 cm	480.07	479.68	94.79
FAD	*Lithraphidites acutus*	U1516C‐34R‐4, 6–8 cm	484.29	U1516C‐35R‐1, 87–90 cm	485.76	485.03	96.16

*Note*. Data for Site U1516 are from Petrizzo et al. (2021). Ages according to Gradstein et al. (2012). FAD = first appearance datum; LAD = last appearance datum; LO = lowest occurrence; HO = highest occurrence; nr = bioevent not reliable; m rCCSF = revised Core Composite depth below Sea Floor in meters.

The interval from 250.05 to 264.02 m rCCSF (Figure 2) consists of alternating light gray nannofossil mudstone and dark gray nannofossil‐bearing claystone. Planktonic foraminifera are common to abundant, moderately to well‐preserved and dominated by small‐sized specimens. Species richness varies from 3 to 7 (Figure 3) and biostratigraphically‐significant taxa are absent. Calcareous nannofossils are moderately to well‐preserved and range from common to abundant. Average species richness in this interval is about 45 taxa (Figure 3).

Specimens of *Gartnerago nanum* and *Gartnerago ponticula* are well represented; however, differentiation between these two species is only possible in better preserved samples. As a result, the last occurrence of both species is used to mark the last appearance datum (LAD) of *G*. *nanum* which occurs at 258.49 m rCCSF (Figure 2, Table 1), indicating an age of approximately 94.8 Ma for this level (Table 1). Moreover, the concurrent range of *G*. *nanum* and *L*. *acutus* indicates the lower part of Subzone CC 10a (Figure 2) corresponding to an early late Cenomanian age (Gradstein et al., 2012). The top of this interval coincides with the LAD of *L*. *acutus* at 250.05 m rCCSF and suggests an age of about 94.4 Ma for this level (Table 1) although the very rare and sporadic occurrence of this species at Site U1513 renders this observation an indicator of the minimum age only.

The interval from 247.52 to 250.05 m rCCSF (Figure 2) records the transition from nannofossil claystone deposition at the base to the organic‐rich claystone characterized by low CaCO_3_ content at the top (244.21–247.52 m rCCSF; Figure 2). Sample U1513A‐45X‐3, 16–18 cm at 247.81 m rCCSF contains the highest occurrence of *C*. *kennedyi*, *Axopodorhabdus biramiculatus*, *Discorhabdus watkinsi*, and *Ceratolithina naturalisteplateauensis*. Sample U1513‐45X‐2, 144–147 cm at 247.60 m rCCSF contains the LAD of *Helenea chiastia* (erroneously spelled *H*. *chiasta* in Petrizzo et al., 2021) indicating a minimum age of 93.9 Ma. This sequence of events correlates well with what was observed at nearby Site U1516 (Petrizzo et al., 2021). The next overlying sample (U1513D‐19R‐3, 46–49 cm) at 247.52 m rCCSF marks the base of the 3.3 m‐thick barren interval with low CaCO_3_ content (Figures 2 and 3). The simultaneous disappearance of *C*. *kennedyi* and *A*. *biramiculatus* at 247.81 m rCCSF could indicate a disconformity between these bioevents and the overlying LAD of *H*. *chiastia* at 247.60 m rCCSF (Figure 2).

According to the age‐depth model and the calculated sedimentation rates at Site U1513 (Figure 3, Table 1) the disconformity between the two bioevents corresponds to a hiatus of about 200 ky, and *C*. *kennedyi* is interpreted to be reworked from older sediments. Samples within the 2 m (242.23–244.21 m rCCSF) above the barren interval (Figure 2) contain poorly preserved, strongly etched calcareous nannofossils, with the most intense diagenetic destruction proximal to the barren zone. Despite the diagenetic alteration, these assemblages have sufficient richness (20–32 species, Figure 3) to allow a confident assignment to Subzone CC 10b, whose base is defined by the LAD of *H*. *chiastia*.

The planktonic foraminiferal assemblages in this interval are still low in diversity, although species richness significantly increases to 12 species 2 m above the barren interval (Figure 3). *Laeviella bentonensis* (“*Globigerinelloides*” *bentonensis* in Petrizzo et al., 2021) is very rare and only sporadicly present at Site U1513. It was last recorded at 242.59 m rCCSF (sample U1513D‐18R‐3, 71–74 cm) within the nannofossil Subzone CC 10b, in disagreement with its record at Site U1516 and at low latitudes where it last occurs in the upper part of Subzone CC 10a. At 242.23 m rCCSF (sample U1513D‐18R‐3, 35–38 cm) the double keeled *Dicarinella hagni* appears within nannofossil Subzone CC 10b, in agreement with the record observed at Site U1516 (Petrizzo et al., 2021).

The overlying succession from 224.50 to 241.78 m rCCSF (Figure 2) consists of nannofossil‐bearing mudstone with moderately preserved nannofossil assemblages in a mudstone matrix. Species richness of calcareous nannofossil assemblages averages about 35 taxa (Figure 3). The late Cenomanian evolution in the polycyclolithinids provides biostratigraphic control for this interval, with the sequential FADs of *Eprolithus octopetalus* (at 241.33 m rCCSF), *Eprolithus moratus* (at 236.65 m rCCSF), and *Quadrum gartneri* (at 227.80 m rCCSF).

The appearance of *Q*. *gartneri* that marks the base of Zone CC 11 and the disappearance of *H*. *chiastia* that defines the base of Subzone CC 10b are both reliable datums for approximating the base of the Turonian Stage in the Global Stratotype Sections and Points (GSSP) type section at Pueblo, Colorado, whose stage criterion is the lowest occurrence of the ammonite *Watinoceras devonense* (Kennedy et al., 2000, 2005). Specifically, *Q*. *gartneri* first occurs slightly above the stage criterion (Tsikos et al., 2004) and *H*. *chiastia* is documented in the same interval recording *W*. *devonense* (Corbett et al., 2014; Kennedy et al., 2005). In this study we tentatively place the Cenomanian/Turonian boundary at the highest occurrence of *H*. *chiastia* following Gradstein et al. (2012, 2020) and Corbett et al. (2014) although we cannot exclude that its topmost stratigraphic range could fall within the barren interval with low CaCO_3_ content.

Planktonic foraminifera species richness is slightly higher than in the subjacent interval (average of 8 species, Figure 3) and specimens are moderately to well preserved. However, no age diagnostic taxa (i.e., *R*. *cushmani*, *Helvetoglobotruncana helvetica*) useful to constrain the Cenomanian/Turonian boundary have been found similar to the record at Site U1516 (Petrizzo et al., 2021). Accumulation rates for this interval are 47.8 m/m.y. (Figure 3).

Because of the absence of marker species and the possible diachroneity of the occurring planktonic foraminiferal species, the *R*. *cushmani*, the *Whiteinella archaeocretacea* and the *T*. *greenhornensis* Zones (Figure 2) are identified according to their stratigraphic position and equivalence with the low latitude biozones (Petrizzo & Gilardoni, 2020; Robaszynski & Caron, 1995) and are constrained by correlation with calcareous nannofossils events.

### 3.3 Composition of the Microfossil Assemblages

**Figure 4:**
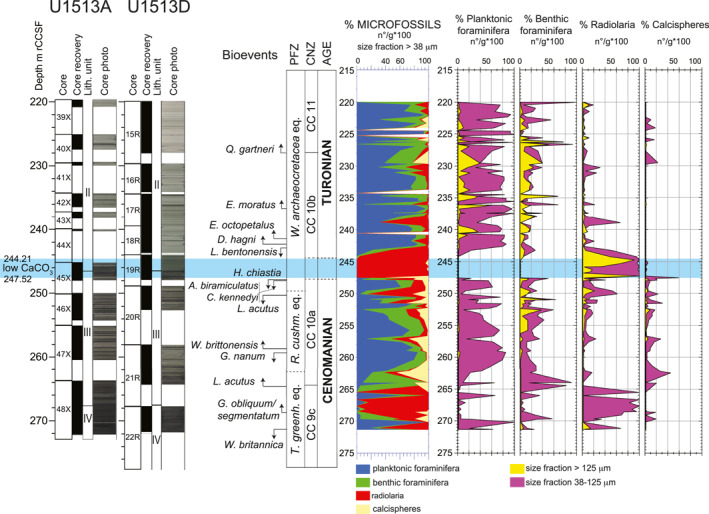
Holes U1513A and U1513D. Core recovery, lithologic units, core photos according to Huber et al. (2019a). Biostratigraphy and age according to this study. Microfossils (planktonic foraminifera, benthic foraminifera, radiolaria and calcispheres) absolute abundance (number of specimens per gram of dry sediment = n°/g) are calculated in the >38 μm size fraction and expressed in percentage. Abbreviations: m rCCSF = revised Core Composite depth below Sea Floor in meters; PFZ = Planktonic Foraminifera Zones; CNZ = Calcareous Nannofossils Zones; *W*. *archaeocretacea* eq. = *Whiteinella archaeocretacea* equivalent; *R*. *cushm*. eq. = *Rotalipora cushmani* equivalent; *T*. *greenh*. eq. = *Thalmanninella greenhornensis* equivalent.

The microfossil assemblages at Site U1513 are characterized by the dominance of small‐sized (38–125 μm) specimens whereas large‐sized specimens (>125 μm) are always a minor component. In general, planktonic foraminifera dominate assemblages throughout except for the intervals where radiolaria comprise nearly the total microfossil assemblages (Figure 4).

**Figure 5:**
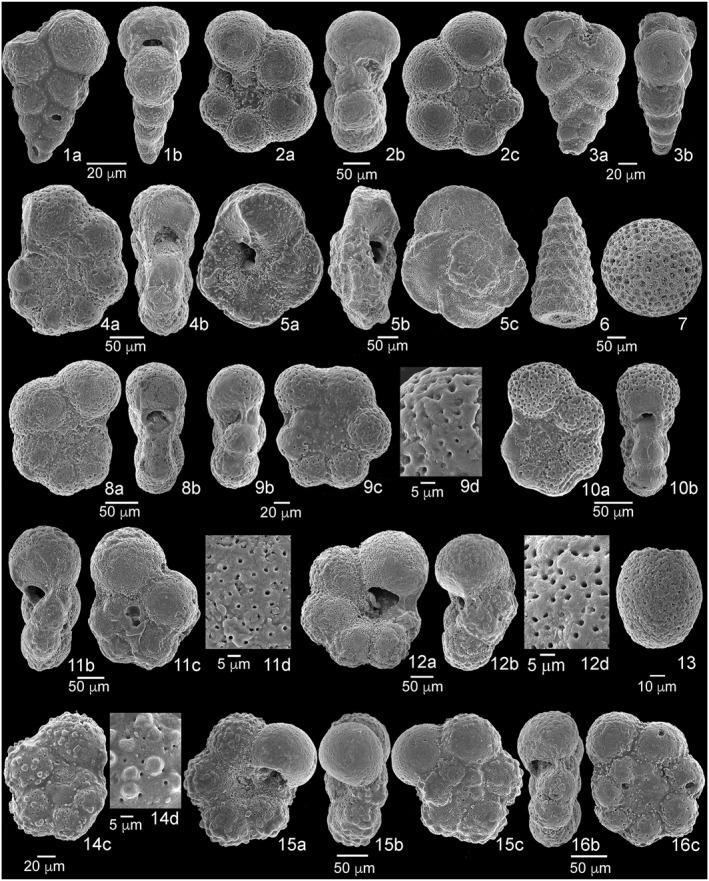
Scanning electron microscope (SEM) images of microfossils. 1a‐b, *Protoheterohelix washitensis*, sample 369‐U1513D‐16R‐1, 30–33 cm. 2a–c, *Muricohedbergella planispira*, sample 369‐U1513D‐16R‐3, 95–98 cm. 3a–b, *Planoheterohelix moremani*, sample 369‐U1513D‐16R‐3, 95–98 cm. 4a–b, *Planohedbergella ultramicra*, sample 369‐U1513D‐16R‐4, 95–98 cm. 5a–c, *Dicarinella canaliculata*, sample 369‐U1513D‐18R‐2, 45–48 cm. 6, Nassellaria Radiolaria, sample 369‐U1513D‐19R‐1, 90–93 cm. 7, Spumellaria Radiolaria, sample 369‐U1513D‐19R‐1, 90–93 cm. 8a–b, *Laeviella bentonensis*, sample 369‐U1513D‐18R‐3, 71–74 cm. 9b–d, *Microhedbergella praeplanispira*, sample 369‐U1513A‐46X‐1, 95–98 cm. 10a–b, *Laeviella bentonensis*, sample 369‐U1513A‐47X‐1, 48–51 cm. 11c–d, *Microhedbergella albiana*, sample 369‐U1513A‐47X‐2, 135–138 cm. 12a–b, d, *Whiteinella brittonensis*, sample 369‐U1513D‐21R‐1, 100–103 cm. 13, calcisphera, sample 369‐U1513A‐48X‐1, 65–68 cm. 14c–d, *Muricohedbergella delrioensis*, sample 369‐U1513D‐21R‐3, 95–98 cm. 15a–c, *Muricohedbergella delrioensis*, sample 369‐U1513A‐48X‐1, 65–68 cm. 16b–c, *Microhedbergella praeplanispira*, sample 369‐U1513A‐48X‐3, 35–38 cm. a, umbilical view; b, side view; c, spiral view; d, detail of the wall.

The stratigraphic interval from 265.52 to 271.31 m rCCSF (Figure 4) is mainly composed of small‐sized radiolaria ranging from 20% to 100% of microfossils in the >38 μm fraction and is characterized by the dominance of specimens belonging to the order Spumellaria (Petrizzo, Amaglio, et al., 2022). Planktonic foraminifera are rare and show two peaks in abundance near the base and near the top of this interval. The latter coincides with the increase in abundance of benthic foraminifera although benthic foraminifera continue to have low abundance values. Calcispheres are subordinate in abundance but comprise up to the 10% of the total microfossil assemblages in a few samples. Foraminiferal preservation ranges from good to moderate as some tests show no infilling, although some evidence of secondary alteration is visible (Figure 5). Calcareous nannofossils from this interval are generally poorly preserved; although, two samples within the interval with moderate preservation have relatively rich (>40 species) assemblages (Petrizzo, Amaglio, et al., 2022).

The overlying interval from 247.60 to 264.47 m rCCSF (Figure 4) is dominated by small‐sized planktonic foraminifera that decrease slightly in abundance upward, whereas small and large‐sized radiolaria increase in abundance upward. Small‐sized (38–125 μm) benthic foraminifera are abundant at the base of the interval (80%–90% of the total microfossils) then show a slight decrease in abundance coincident with the consistent occurrence of large‐sized (>125 μm) benthic specimens. Calcispheres show a maximum peak in abundance (44% of the total microfossils) in the lower part of the interval at 261.41 m rCCSF (Figure 4). Preservation of foraminifera is good as most tests show no infilling and minor evidence of secondary recrystallization (Figure 5). Calcareous nannofossils in this interval are abundant to common and generally well‐preserved, with consistently rich (average > 43 species) assemblages (Petrizzo, Amaglio, et al., 2022).

The interval of low carbonate content (from 247.52 to 244.21 m rCCSF; Figure 4) is dominated by radiolaria throughout with the exception of a peak in calcispheres registered in the first sample devoid of calcareous plankton at 247.52 m rCCSF (Petrizzo, Amaglio, et al., 2022). Radiolaria in this interval are small‐sized in the lower part whereas large‐sized specimens dominate in the upper part of the interval. The number of Nassellaria specimens show the highest abundance recorded at Site U1513 (Petrizzo, Amaglio, et al., 2022).

The interval from 243.21 to 219.97 m rCCSF (Figure 4) is dominated by planktonic foraminifera that display cyclic fluctuations in absolute abundance anti‐phased to the absolute abundance of benthic foraminifera. Radiolaria and calcispheres rank next in abundance, averaging 10%–15% of the microfossil assemblage. Four samples at 224.39, 225.25, 234.30, and 240.55 m rCCSF are barren of microfossils (Figure 4; Petrizzo, Amaglio, et al., 2022). Some of the foraminifera in this interval have test walls showing moderate preservation as they have coarser calcite overgrowths and are infilled with sparry calcite (Figure 5). Calcareous nannofossils are common and moderately preserved in this interval, with average species richness of about 28 reflecting the reduction in taxa following the Cenomanian‐Turonian extinction (Petrizzo, Amaglio, et al., 2022).

### 3.4 Benthic Foraminiferal Assemblages

Benthic foraminifera show a consistent stratigraphic distribution at Site U1513 (Figure 4; Petrizzo, Amaglio, et al., 2022) and are generally common in the small‐sized fractions (38–125 μm). Benthic foraminiferal individuals in these fractions could not be confidently identified at the genus or species level because the assemblages are mostly composed of juvenile specimens. For taxa that could be confidently identified, the assemblages are dominated by bathyal taxa that predominantly show depth ranges of 400–2,000 m (i.e., Kaminski & Gradstein, 2005; Murray, 2006; Widmark & Speijer, 1997).

Calcareous taxa are more abundant whereas agglutinated taxa represent only a minor component and never exceed 10% of the total assemblages. The most abundant taxa throughout the studied interval are gavelinellids (including *Stensioeina* and *Gavelinella*), *Gyroidinoides*, and *Clavulinoides*. *Praebulimina* is common only from above the interval of low CaCO_3_ content.

The biostratigraphically diagnostic benthic foraminifera *Gavelinella intermedia*, *Gavelinella schloenbachi*, and *Scheibnerova protoindica*, which have been reported to occur across the Cenomanian‐Turonian boundary interval in southern high latitudes localities (Hornibrook et al., 1989; Scheibnerová, 1976) were also identified. Diversity throughout the studied interval averages 70 species and is quite low compared to the 100–200 taxa recorded in the Turonian to Santonian stratigraphic interval at Site U1513 (Petrizzo, MacLeod, et al., 2022; Wolfgring et al., 2022). Though no extinctions of benthic foraminifera are evident, a shift in dominant benthic foraminiferal taxa in the upper part of the OAE 2 interval is observed.

A detailed study of the benthic foraminiferal assemblages at sites U1513 and U1516 focused on their response to the paleonvironmental perturbation in the bottom waters during the OAE 2 will be the subject of future publications.

### 3.5 Planktonic Foraminiferal Assemblages

**Figure 6:**
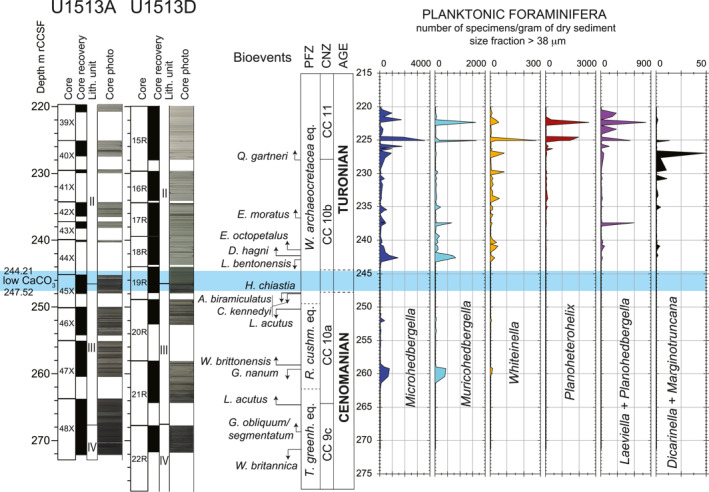
Holes U1513A and U1513D. Core recovery, lithologic units, core photos according to Huber et al. (2019a). Biostratigraphy and age according to this study. Planktonic foraminifera absolute abundance (number of specimens per gram of dry sediment = n°/g) are calculated in the >38 μm size fraction. Abbreviations: m rCCSF = revised Core Composite depth below Sea Floor in meters; PFZ = Planktonic Foraminifera Zones; CNZ = Calcareous Nannofossils Zones; *W*. *archaeocretacea* eq. = *Whiteinella archaeocretacea* equivalent; *R*. *cushm*. eq. = *Rotalipora cushmani* equivalent; *T*. *greenh*. eq. = *Thalmanninella greenhornensis* equivalent.

The composition of the planktonic foraminiferal assemblages at Site U1513 (Figure 6; Petrizzo, Amaglio, et al., 2022) is similar to those observed at Site U1516 (Petrizzo et al., 2021). They are characterized by low species diversity (Figure 3) and by the absence of the typical Cenomanian‐Turonian genera *Rotalipora*, *Thalmanninella*, and *Helvetoglobotruncana* while *Praeglobotruncana* and the keeled genera *Dicarinella* and *Marginotruncana* occur with few species compared to the assemblages of the equivalent stratigraphic interval at mid‐low latitudes (e.g., Eastbourne, England: Falzoni & Petrizzo, 2020; Falzoni et al., 2018; Hart et al., 2002; Keller et al., 2001; Paul et al., 1999; Vocontian Basin: Falzoni, Petrizzo, Clarke, et al., 2016; Falzoni, Petrizzo, Jenkyns, et al., 2016; Grosheny et al., 2006, 2017; Spain: Lamolda et al., 1997; Austria: Gebhardt et al., 2010; Switzerland: Strasser et al., 2001; Westermann et al., 2010; Umbria‐Marche Basin, Italy: Coccioni & Luciani, 2005; Coccioni & Premoli Silva, 2015; Luciani & Cobianchi, 1999; Mort et al., 2007; Premoli Silva & Sliter, 1995; Scopelliti et al., 2004; Tarfaya, Morocco: Falzoni et al., 2018; Keller et al., 2008; Tunisia: Caron et al., 2006; Nederbragt & Fiorentino, 1999; Reolid et al., 2015; Robaszynski et al., 1990, 1993; Tibet: Bomou et al., 2013; Blake Nose, NW Atlantic: Huber et al., 1999; Japan: Hasegawa, 1999; Western Interior Seaway, US: Caron et al., 2006; Desmares et al., 2007; Eicher & Diner, 1985; Elderbak & Leckie, 2016; Eicher & Worstell, 1970; Keller & Pardo, 2004; Leckie, 1985; Leckie et al., 1998; Lowery & Leckie, 2017, among many others).

The distribution and abundances of the planktonic foraminiferal species occurring at Site U1513 are reported in Petrizzo, Amaglio, et al. (2022). The assemblages are mainly composed of small‐sized (38–125 μm) specimens of *Microhedbergella praeplanispira*, *Microhedbergella pseudodelrioensis*, *Muricohedbergella delrioensis*, *Muricohedbergella planispira*, *Whiteinella baltica*, *Planoheterohelix* sp., and *Planohedbergella ultramicra*. Larger specimens show a scattered occurrence and are represented by *Whiteinella brittonensis*, *D*. *hagni*, *Marginotruncana coldreriensis*, *Marginotruncana caronae*, *L*. *bentonensis* and *Praeglobotruncana stephani*. The latter two species are recorded in low numbers and were found in only four samples.

Planktonic foraminifera are rare from 265.52 to 271.31 m rCCSF (Figure 6) and species diversity ranges from 2 to 3 (Figure 3). *Microhedbergella* and *Muricohedbergella* are the sole genera occurring with the former displaying maximum values of 6 and 4 specimens per gram of dry sediment near the base and near the top of this interval, respectively (Figure 6).

The overlying interval from 247.60 to 264.47 m rCCSF (Figure 6) is characterized by an increase in number of species ranging from 3 to 7 (Figure 3). The assemblage is dominated by the small‐sized *Microhedbergella* that reach 743 specimens per gram of dry sediment at 259.20 m rCCSF. *Muricohedbergella delrioensis* shows a continuous record but never exceed 416 specimens per gram of dry sediments. *Whiteinella*, *Planoheterohelix*, and the planispiral taxa (*Laeviella* and *Planohedbergella*) display a scattered distribution and low number of specimens ranging from 1 to 12 specimens per gram of dry sediment whereas the keeled *Dicarinella* and *Marginotruncana* are absent.

Planktonic foraminifera are totally absent in the interval of low carbonate content (from 244.21 to 247.52 m rCCSF) and become common in the overlying interval from 219.97 to 243.21 m rCCSF (Figure 6) where species diversity increases to 15 in some levels (Figure 3). *Microhedbergella* is still the dominant genus. It ranges from about 20 to 900 specimens per gram of dry sediments with a peak value close to 3,600 specimens per gram of dry sediments at 225.01 m rCCSF. *Muricohedbergella* is the next most common genus varying from 5 to 1,653 specimens per gram of dry sediment. *Whiteinella* ranges from 2 to 70 specimens per gram of dry sediments and increase to 276 specimens at 225.01 m rCCSF. *Planoheterohelix* ranges from 3 to 2,603 specimens per gram of dry sediment whereas *Planohedbergella* ranges from 1 to 813 specimens per gram of dry sediment. The keeled *Dicarinella* and *Marginotruncana* show an almost continue stratigraphic distribution but never exceed 50 specimens per gram of dry sediments (Figure 6).

## 4 Discussion: Comparison Between Sites U1513 and U1516

### 4.1 Correlation and Identification of OAE 2

**Figure 7:**
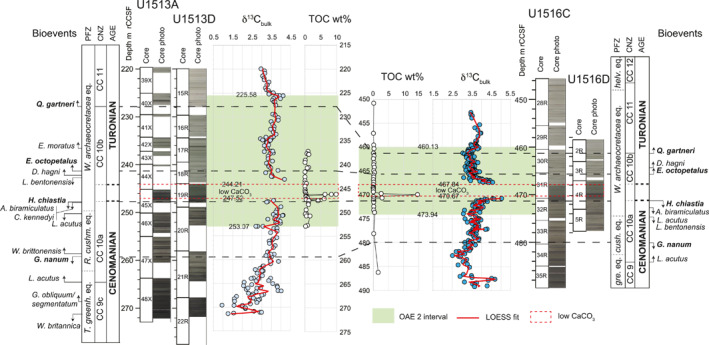
Stratigraphic correlation between sites U1513 and U1516. Site U1513: core recovery and core photo from Huber et al. (2019a); planktonic foraminifera and calcareous nannofossil biostratigraphy, age, carbon isotope bulk carbonate, interval of low CaCO_3_ content and total organic carbon (TOC) according to this study (see Figure 2). Site U1516: data are from Petrizzo et al. (2021) except planktonic foraminifera Zones and the position of the Cenomanian/Turonian boundary that have been revised in this study (see text for explanation). For the identification of the OAE 2 interval (light green band) see explanation in the text. Abbreviations: m rCCSF = revised Core Composite depth below Sea Floor in meters; PFZ = Planktonic Foraminifera Zones; CNZ = Calcareous Nannofossils Zones; *helv*. eq = *Helvetoglobotruncana helvetica* equivalent; *W*. *archaeocretacea* eq. = *Whiteinella archaeocretacea* equivalent; *R*. *cushm*. eq., *cush*. eq. = *Rotalipora cushmani* equivalent; *T*. *greenh*. eq., *gre*. eq. = *Thalmanninella greenhornensis* equivalent.

**Figure 8:**
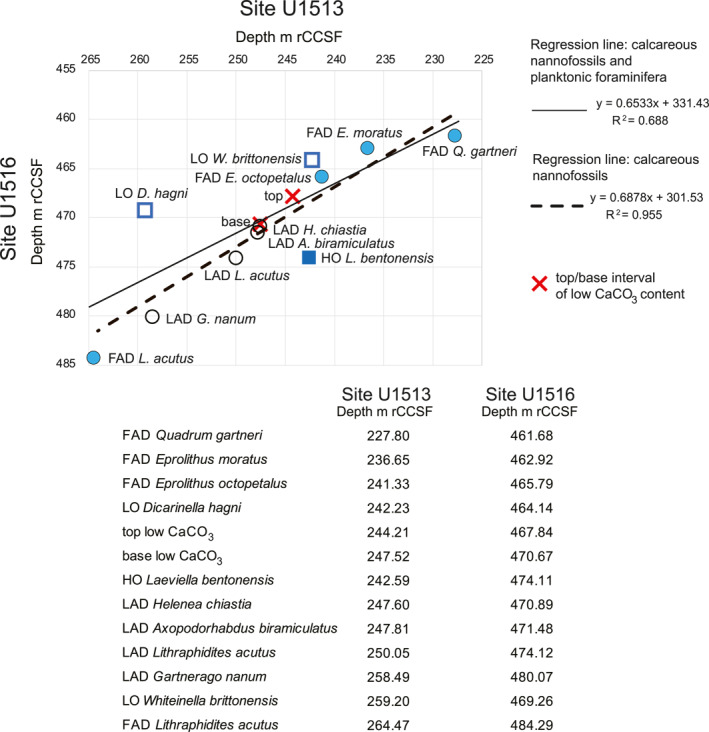
Graphic correlation of the shared bioevents and the position of the top and base of the interval of low carbonate content. See text for the explanation of the best‐fit regression lines. Circles are calcareous nannofossils, squares are planktonic foraminifera. Abbreviations: FAD = first appearance datum; LAD last appearance datum; LO = lowest occurrence; HO = highest occurrence; m rCCSF = revised Core Composite depth below Sea Floor in meters.

The correlation between sites U1513 and U1516 (Figure 7) is pinned at the highest TOC values registered within the organic‐rich, black claystone layer characterized by low CaCO_3_ content supported by the biostratigraphic events (Figure 8, Table 1) and the δ^13^C values. The interval of low CaCO_3_ content (Figure 7) is 3.31 m thick at Site U1513 (in cores U1513A‐45X‐2 and U1513D‐19R‐2) and 2.83 m thick at Site U1516 (in cores U1516C‐31R‐4 and U1516D‐4R‐3).

The calcareous nannofossil assemblages from sites U1513 and U1516 record similar, but not identical, histories. The lower portions of the successions at Site U1513 (interval between LAD *W*. *britannica* and FAD *L*. *acutus*, equivalent to the upper Subzone CC 9c) are characterized by sparse (<5% of sediment volume) and poorly preserved assemblages with generally low species richness (in average 16–19 species at both sites). Preservation improves and abundance increases at both sites starting from the base of Subzone CC 10a, and the interval from the FAD of *L*. *acutus* to the base of the interval with low CaCO_3_ content (Figure 7) contains the highest species richness values (in average 42‐43 species at both sites).

Although the sites are 69 km apart and are not separated by any obvious physiographic barriers, there are substantial differences in the composition of the assemblages in the two sites for this interval. For example, *C*. *kennedyi*, a well‐documented tropical to temperate biomarker, is present, and sometimes more than 1% in abundance, in more than half of the samples (9 out of 16 samples; Petrizzo, Amaglio, et al., 2022) in this interval at Site U1513, whereas it was only observed as a single specimen in one sample in the equivalent interval at Site U1516. Above the interval of low CaCO_3_ content in Subzone CC 10b, the calcareous nannofossils from both sites become again similar in composition and richness (in average 35–37 species at both sites) and exhibit the same sequence of bioevents as in coeval tropical to temperate assemblages.

In general, the planktonic foraminiferal assemblages at Site U1513 are similar to those documented at Site U1516 (Petrizzo et al., 2021) although species that should be useful for intra basin correlation showed surprising differences between sites. For instance, at Site U1516 the highest occurrence of *L*. *bentonensis* precedes the LAD of *A*. *biramiculatus*, whereas the opposite is observed at Site U1513. Similarly, the lowest occurrence of *D*. *hagni* is recorded above the FAD of *E*. *octopetalus* at Site U1516, whereas at Site U1513 that sequence of events is inverted (Figure 7). The observed inversions in the sequence of calcareous nannofossil and planktonic foraminiferal events suggest that they are very close in age and the inversions might be related to differences in the preservation of the specimens and/or to the small size of the core samples that prevent collecting a sufficient number of foraminiferal specimens to accurately record the distribution of relatively rare taxa.

The best fit graphic correlation regression lines (Figure 8) using the shared calcareous plankton bioevents and the position of the top and base of the interval of low CaCO_3_ content enables precise correlation between the two sites. The reliability of the sequence of the calcareous nannofossils events for correlation is well supported as events consistently align along the best‐fit regression line (correlation coefficient 0.955; Figure 8), whereas planktonic foraminifera show a low degree of correlation and often plot markedly off the regression line. Although the planktonic foraminiferal bioevents are delayed or anticipated between sites (i.e., *D*. *hagni*, *L*. *bentonensis*, *W*. *brittonensis*) the correlation coefficient of 0.688 for the combined calcareous plankton groups indicates a reasonable consistency (Figure 8).

The OAE 2 interval at Site U1516 (Figure 7; see also Petrizzo et al., 2021) is identified from the onset of the positive δ^13^C trend at the base (473.94 m rCCSF) to the first positive shift in δ^13^C values at the top (460.13 m rCCSF) just above an interval of lower δ^13^C values based on correlation with the low latitude Eastbourne section in England and according to the OAE 2 definition by Jarvis et al. (2011), Gambacorta et al. (2015), and Jenkyns et al. (2017). Precise determination of the onset of the OAE 2 interval at Site U1513 is complicated by the absence of peak positive excursion values which suggests the low CaCO_3_ content interval has a greater chronostratigraphic extent at Site U1513 compared to Site U1516. Nevertheless, the base of OAE 2 at Site U1513 is tentatively placed at the lowest δ^13^C value within the increasing trend of the δ^13^C values at 253.07 m rCCSF (Figure 7), whereas the termination of the OAE 2 interval (Figure 7) is based on comparison to Site U1516 and is placed at 225.58 m rCCSF, a level that marks where δ^13^C values are highest before a progressive decrease from previous high values.

### 4.2 Similarities and Discrepancies Between Sites

**Figure 9:**
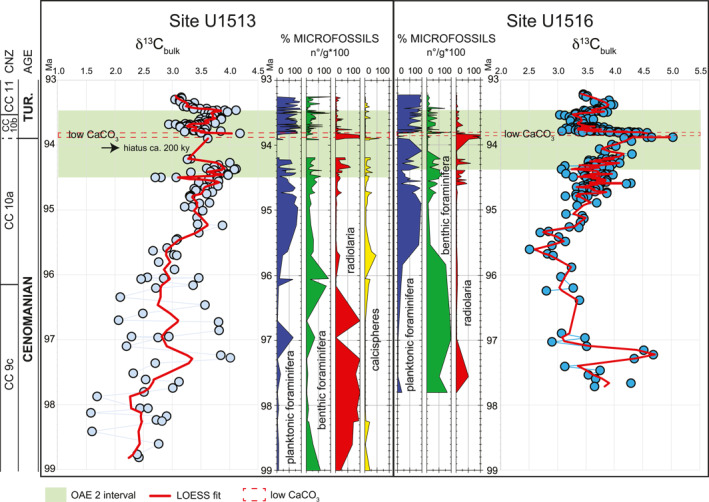
Correlation of the microfossil abundances and δ^13^C record between sites U1513 and U1516 in the interval from 93.2 to 99.0 Ma. Age scale derived from the age‐depth model in Figure 3 and Table 1. The Cenomanian/Turonian boundary is placed at 93.9 Ma (Meyers et al., 2012; see explanation in the text). Microfossils absolute abundance (number of specimens per gram of dry sediment = n°/g) are calculated in the >38 μm size fraction and expressed in percentage. CNZ = calcareous nannofossils Zones.

**Figure 10:**
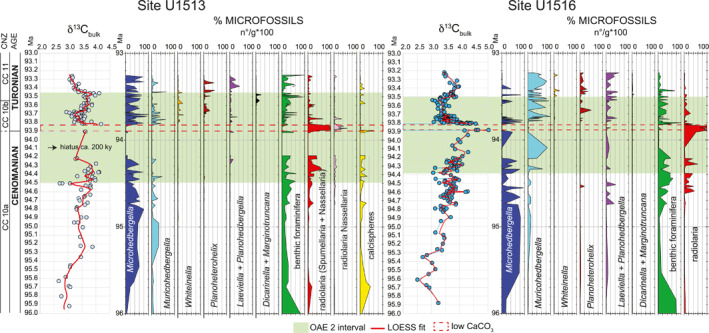
Carbon isotope bulk carbonate and abundances of planktonic foraminifera genera, benthic foraminifera, radiolaria and calcispheres at sites U1513 and U1516 in the interval from 93.2 to 96.0 Ma. Age scale according to the age‐depth model in Figure 3 and Table 1. The Cenomanian/Turonian boundary is placed at 93.9 Ma (Meyers et al., 2012; see explanation in the text). Microfossils absolute abundance (number of specimens per gram of dry sediment = n°/g) are calculated in the >38 μm size fraction and expressed in percentage. CNZ = calcareous nannofossils zones.

Carbon isotope data and microfossil distributions and abundances are plotted on a common age scale to highlight similarities and discrepancies between sites U1513 and U1516 (Figure 9). Age estimates are derived from the age model presented in Figure 3 and Table 1. Ages for the bioevents are cited from published sources (Gradstein et al., 2012) and are considered only as estimates since none of the bioevents have been directly calibrated using radiometric ages. In this study the Cenomanian/Turonian boundary is approximated at the LAD of *H*. *chiastia* (Table 1) and placed within the barren interval of low CaCO_3_ content (because of the uncertainty in the identification of the topmost range of *H*. *chiastia*) in Figures 2, 4, 6, and 7, whereas in Figures 9 and 10 the boundary is placed at 93.9 Ma according to the intercalibrated astrochronologic and radioisotopic time scale developed for the Cenomanian‐Turonian boundary interval near the GSSP in Colorado (Meyers et al., 2012).

The mismatch observed in the age estimates of the base and top of OAE 2 between sites are minor, estimated as 0.13 my for the base and 0.04 my for the top, and they are likely related to the sampling resolution and to the uncertainty from using mean depths of the bioevents, as shown in the age‐depth model (Table 1, Figure 3). The duration of OAE 2 in the Mentelle Basin is estimated as 950 ky. Bio‐chronostratigraphic data reveal the occurrence of a short hiatus of about 200 ky at a disconformity about 29 cm below the interval of low CaCO_3_ content, which might result from erosion of sediments by seafloor currents and winnowing. This observation is consistent with the sharp contact between the gray and black clay‐rich claystone observed in core U1513A‐45X‐2, 137 cm (Huber et al., 2019a). The OAE 2 interval lacks a clear positive δ^13^C excursion in the bulk carbonate record at Site U1513 which likely results from the non‐preservation of CaCO_3_ in sediments representing the time of the δ^13^C excursion.

The earlier part of the studied interval (95.7–99.0 Ma) seems remarkably different between sites both in the carbon isotope record and in the composition of the microfossil assemblages (Figure 9). The δ^13^C values at Site U1513 show large fluctuations of 2‰ among samples from 96.0 to 99.0 Ma. In contrast, at Site U1516 there is one 2‰ excursion from ∼97.0–97.5 Ma and a second single point excursion at 97.7 Ma, but values otherwise fluctuate by only about 0.5‰ through the rest of the lower part of the section (Petrizzo, Amaglio, et al., 2022). At Site U1513 radiolaria are the main component of the microfossil assemblages in this interval whereas planktonic and benthic foraminifera occur in few samples and become dominant only in the upper part of the interval. Calcispheres also increase in abundance in the upper part of the interval and reach their maximum abundance at 95.7 Ma where they comprise 40% of the total microfossils. In contrast, at Site U1516 radiolaria only occur in the lower part of the interval and the microfossil assemblages are dominated by benthic foraminifera (Figure 9).

The interval from 94.8 to 95.7 Ma is characterized by the dominance of planktonic foraminifera at both sites. Benthic foraminifera are subordinate in abundance in this interval, and radiolaria never exceed 20%–25%. Calcispheres, which vary from 5% to 30%, are only observed at Site U1513 (Figure 9). At both sites the overlying interval (94.2 to 94.8 Ma) registers a decrease in planktonic foraminifera that alternates in abundance with the other microfossils. The interval from 93.9 to 94.2 Ma contains the hiatus at Site U1513 whereas at Site U1516 planktonic foraminifera alternate in abundance with radiolaria (Figure 9). The interval of low CaCO_3_ content (93.8–93.9 Ma) is characterized by the dominance of radiolaria with the exception of three samples in the middle part of the interval that contain calcispheres (up to 5% of the total microfossils) at Site U1513 and foraminifera (up to 30% of the total microfossils) at Site U1516 (Figure 9; Petrizzo, Amaglio, et al., 2022; Petrizzo et al., 2021). Planktonic foraminifera are the dominant microfossils throughout the interval from 93.2 to 93.8 Ma at both sites although they are less abundant at Site U1513 than at Site U1516 (Figure 9).

The main discrepancies between sites U1513 and U1516 are observed in the lower part of the studied interval (95.7–97.8 Ma) and above the interval of low CaCO_3_ content (Figure 9). In the Cenomanian interval from 95.7 to 97.8 Ma the carbon isotope record and the abundance of foraminifera and radiolaria are different between sites hampering identification of comparable patterns. The differences are likely attributable to variations in marine productivity and sediment provenance. The dominance of radiolaria and the sporadic occurrence of only small‐sized planktonic and benthic foraminifera at Site U1513 certainly indicates high surface water fertility that may have inhibited foraminifera populations. The high amount of biogenic silica could be explained by either an increase of the terrigenous influx and associated enhanced delivery of continental nutrients (Wagner et al., 2004) from the nearby southwest Australian margin (Perth Basin and Leeuwin Block: Chen et al., 2022) or by nutrient‐rich volcanogenic detritus derived from the volcanism of the Central Kerguelen Plateau (Coffin et al., 2002; Jiang et al., 2021) likely transported by the westerly winds and surface ocean currents. However, additional geochemical and clay mineralogy proxy data are needed to reconstruct the sediment provenance in the Mentelle Basin and explain the differences between sites U1513 and U1516 observed in the late Cenomanian record.

Sedimentation rates of equivalent stratigraphic intervals identified according to the calcareous nannofossil datums are similar between the sites below the interval of low CaCO_3_ content, but they are significantly different in the interval from 93.5 to 93.9 Ma (Figure 9) between the LAD of *H*. *chiastia* and the FAD of *Q*. *gartneri* (Figure 7). At Site U1513 the estimated average rate is 47.8 m/m.y. as compared to an average rate of 20.4 m/m.y. at Site U1516 (Figure 3, Table 2). The difference in sedimentation rates observed between sites from above the interval of low CaCO_3_ content and up‐section might be explained by the higher carbonate preservation in the water column at Site U1513 as revealed by the CaCO_3_ content that varies from 30% to 67% (Figure 2), whereas it ranges from 10% to 49% at Site U1516 (Figure 5 in Petrizzo et al., 2021). This observation suggests a greater paleodepth at Site U1513 than Site U1516 and is compatible with their inferred paleogeographic positions. Specifically, Site U1513 was located on the northwest margin of a structural high in the Mentelle Basin (Huber et al., 2019a) and likely more influenced by oceanic circulation in the nearby Perth Abyssal Basin compared to Site U1516, which was located on the southwest margin of the structural high and closer to both the Antarctic and Australian continental margin (Figure 1). Therefore, Site U1513 could be inferred to represent a greater depth than Site U1516, although additional data are needed to support this hypothesis.

**Table 2:** Comparison of the Sedimentation Rates Between Sites U1513 and U1516

	SITE U1513		SITE U1516	
Stratigraphic interval	Sedimentation Rate	Thickness	Sedimentation Rate	Thickness
	m/m.y.	m	m/m.y.	m
FAD *Q. gartneri* to FAD *E. octopetalus*	47.8	12.92	18.5	4.37
FAD *E. octopetalus* to LAD *H. chiastia*	47.8	3.85	22.4	2.11
Low CaCO_3_		3.31		2.83
LAD *H. chiastia* to FAD *G. nanum*	13.6	12.16	12.6	11.34

Remarkable among the microfossil assemblages is the occurrence of common calcispheres at Site U1513 but only sporadic occurrences of these microfossils at Site U1516 where they never exceed abundance values of 1% of the total microfossil assemblages. Presence of abundant calcispheres is linked to intense primary productivity due to eutrophication of the surface waters (Wendler et al., 2002; Wilmsen, 2003), and are interpreted as thermophilic plankton ranging from shelf to shallow bathyal environments (Dias‐Brito, 2000). An acme of pithonellid calcispheres associated with opportunistic planktonic foraminifera has been reported from several stratigraphic sections across the Cenomanian‐Turonian boundary interval and has been interpreted as reflecting a regime with high productivity (e.g., Gale et al., 2000; Hart, 1991; Omana et al., 2014; Pearce et al., 2009; Wendler et al., 2010; Wilkinson, 2011). The rarity of calcispheres at Site U1516 and their consistent occurrence at Site U1513 is intriguing and could reflect their preference for a more open marine setting with a thicker surface mixed layer rich in nutrients. Abundant calcispheres at Site U1513 might also coincide with phases of wind‐driven upwelling or topography‐influenced upwelling of cooler and nutrient‐rich water that moved from deep water toward the ocean surface. Moreover, the occurrence of common tropical to temperate calcareous nannofossils species at Site U1513 and their rarity at Site U1516 further suggests that the two sites were influenced by different water masses.

## 5 Paleoceanographic Interpretations Across OAE 2

The paleoceanographic conditions of the water column across the OAE 2 interval from 93.2 to 96.0 Ma at sites U1513 and U1516 (Figure 10) are interpreted according to the paleoecological preferences of the most common genera of planktonic foraminifera (biserial *Planoheterohelix*, planispiral *Laeviella* and *Planohedbergella*, trochospiral, non‐keeled *Whiteinella*, *Microhedbergella*, *Muricohedbergella*, and keeled *Dicarinella* and *Marginotruncana*), and the co‐occurring benthic foraminifera, radiolaria and calcispheres.

Here we summarize the inferred depth ecologies of each genus used to derive the paleoceanographic interpretation following Petrizzo et al. (2021), who discussed the interspecific patterns of offsets in δ^18^O and δ^13^C stable isotope ratios at Site U1516, and the information on the planktonic foraminiferal paleoecology derived from the literature (Abramovich et al., 2003; Ando et al., 2010; Bornemann & Norris, 2007; Coccioni & Luciani, 2004; D'Hondt & Arthur, 1995; Falzoni et al., 2013, 2014; Falzoni, Petrizzo, Clarke, et al., 2016 ; Hart, 1980, 1999; Huber et al., 1995, 1999; Keller et al., 2001; Leckie, 1987; MacLeod et al., 2001, 2013; Petrizzo et al., 2008, 2017, 2020; Premoli Silva & Sliter, 1999; Wendler et al., 2013; Wilson et al., 2002).

Small‐sized (38–125 μm) *Microhedbergella* were opportunistic taxa interpreted to have lived in the lower mixed layer or in the seasonal thermocline, and they tolerated cooler and productive environments rich in nutrients including area of vertical mixing and upwelling. *Muricohedbergella* and planispiral taxa are interpreted as intermediate species because they show isotopic signatures indicating they either lived in the mixed layer or in the seasonal thermocline. The depth ecology of *Planoheterohelix* is still not clear as it showed adaptation to a wide range of habitats and water mass conditions from the mixed layer to the seasonal and permanent thermocline at different latitudes. In general, biserials are thought to be opportunistic taxa that may proliferate in high productivity and low oxygen conditions; in the Western Interior Seaway their occurrence within OAE 2 is associated with euxinia in the photic zone (Boudinot et al., 2020). *Whiteinella* has been generally associated with a surface/summer and warmer mixed layer habitat. The keeled taxa (*Dicarinella* and *Marginotruncana*) inhabited the thick mixed layer and occupied ecological niches in the surface mixed layer and the thermocline.

Radiolaria, especially Nassellaria, and calcispheres are interpreted to be shallow mixed layer dwellers and are generally associated with conditions of very high fertility in outer shelf to upper slope environments including upwelling regions (De Wever et al., 2003; Dias‐Brito, 2000; Koutsoukos & Hart, 1990; Lisitzin, 1985).

Among benthic foraminifera, gavelinellids are presumably epifaunal taxa interpreted to prefer well oxygenated habitats, but they can tolerate low oxygen levels and benefit from higher food supply (i.e., Gebhardt et al., 2004; Friedrich, 2006). *Gyroidinoides* is an opportunistic taxon with habitat preferences inferred as either infaunal or epifaunal to shallow infaunal and with a varying tolerance for sub‐ and dysoxic conditions, whereas infaunal taxa (*Praebulimina*, *Lenticulina*) and agglutinated taxa tolerated sub‐ to anoxic conditions (Alegret et al., 2003; Friedrich et al., 2006; Jorissen et al., 2007).

### 5.1 Interval Below OAE 2

The lithology below OAE 2 is represented by gray and black clay‐rich claystone (lithostratigraphic Unit III, Huber et al., 2019a) with a carbonate content varying from 20% to 40% (Site U1513: Figure 2; Site U1516: Figure 5 in Petrizzo et al., 2021). In the interval below OAE 2 (Figure 10) planktonic foraminifera dominate and are represented by *Microhedbergella*, which is the most common genus, followed by *Muricohedbergella*. Together these two are almost the only genera occurring in this interval. Planispiral taxa are very rare at Site U1513; they are more common at Site U1516 and reach a peak abundance of about 20% in the upper part of the interval. *Planoheterohelix* is recorded in low numbers only in the upper part of the interval, and the genus is more abundant at Site U1516 than Site U1513. Benthic foraminifera occur throughout the interval but are more common at its base and near the top than they are in the middle portions of the interval. Gavelinellids together with the opportunist taxa *Gyroidinoides*, *Lenticulina*, and *Praebulimina* dominate the benthic assemblages and indicate oxic‐suboxic conditions. Radiolaria show a similar distribution and abundance and are more abundant at the top of the interval. Calcispheres at Site U1513 parallel the distribution pattern of radiolaria but are very rare at Site U1516.

Changes in the composition and abundance of the microfossil assemblages at the two sites are pretty similar and indicate a paleoceanographic setting characterized by reduced water mass stratification resulting from enhanced surface water productivity or vertical mixing. This interpretation is based on the abundance of the opportunistic *Microhedbergella* that alternates with common eutrophic radiolaria in the surface waters. The conditions of very high fertility are more pronounced at the base and top of the interval according to the increased abundance of radiolaria and might have been caused either by small scale variations in sea level that have been estimated to have reached maximum levels of 240–250 m above present‐day mean sea‐level (Haq, 2014; Ray et al., 2019) or alternating phases of enhanced surface water productivity and vertical mixing/upwelling. In addition, the concomitant high abundance of benthic foraminifera with the dominance of gavelinellids and the relatively low values of planktonic foraminifera at the base and top of the interval might suggest episodes of enhanced oxygenation at the sea floor. However, the overall eutrophic feature of the water masses seems more pronounced at Site U1513 because of the rarity of the intermediate dwelling planispiral taxa (*Planohedbergella* and *Laeviella*) and the consistent occurrences of the opportunistic and eutrophic calcispheres.

All together these observations suggest fluctuations in surface water marine productivity and variations in the thickness of the mixed layer likely driven by changes of the nutrient provenance, including either delivery from terrigenous influx from southwest Australia via fluvial input and consequent enhanced delivery of continentally derived nutrients that could have stimulated the marine productivity (Chen et al., 2022) or episodic upwelling of cooler deep water.

### 5.2 OAE 2 Interval

The OAE 2 interval can be subdivided into three subintervals: 1. below, 2. within and above the low CaCO_3_ zone (Figure 10).

1. The lithology in the subinterval below the low CaCO_3_ content interval (Figure 10) is represented by gray and black clay‐rich claystone (lithostratigraphic Unit III: Huber et al., 2019a). The microfossil groups fluctuate in abundance similar to the upper underlying interval, indicating that no substantial biotic changes occurred at the onset of OAE 2, which is here exclusively identified according to the inflection toward lower carbon isotope values. Therefore, we infer a similar paleoceanographic setting characterized by a dominantly eutrophic regime.
  At Site U1513 a hiatus of about 200 ky is inferred near the top of the subinterval based on the age model adopted in this study. The hiatus at Site U1513, which is interpreted as resulting from seafloor erosion, might also represent an interval coinciding with unfavorable conditions for the preservation of pelagic carbonate on the seafloor with waters that were not anoxic enough to allow accumulation of organic matter (Jenkyns, 2010) or to shoaling of the Carbonate Compensation Depth (CCD). A rise in the CCD might have resulted from CaCO_3_ undersaturation of ambient waters due to prolonged ocean acidification during the thermal maximum and the δ^13^C excursion, which have been interpreted to be forced by the high atmospheric pCO_2_ released by volcanic activity associated with the Large Igneous Provinces eruptions (Barclay et al., 2010; Du Vivier et al., 2015; Kuroda et al., 2007) including the Central Kerguelen Plateau LIP (i.e., Jiang et al., 2021; Matsumoto et al., 2022; Robinson et al., 2019). Peak positive values in the δ^13^C record measured at the base of the low CaCO_3_ interval at Site U1516 is instead not registered at Site U1513 as it falls within the time of deposition of the low carbonate sediments (Figure 10).
2. The subinterval with low CaCO_3_ content (Figure 10) is marked by the near absence of calcareous planktonic and benthic foraminifera, lack of other biogenic carbonate, and abundance of siliceous and organic matter‐rich sediments. In this subinterval radiolaria are the sole microfossils present except for a couple of isolated samples that contain two‐three benthic agglutinated and one calcareous benthic foraminifera at Site U1516 and calcispheres at Site U1513.
  The high abundance of radiolaria and the rarity of calcareous microfossils has been previously interpreted (Petrizzo et al., 2021) as indicating very high fertility conditions and, possibly, shoaling of the CCD. Dissolution/absence of calcareous tests agrees with the strong oxidation of organic matter although survival of foraminifera could also have been prevented by a very high surface water fertility leading to expansion of anoxia at the seafloor in agreement with the maximum TOC values registered in this subinterval (Figure 7). The dominance of shallow water nassellarian radiolaria at Site U1513 and Site U1516 (Figure 10) is similar to the record from within the organic‐matter rich deposits of the Bonarelli level of the Italian section in the Umbria‐Marche area (Bąk, 2011; Erbacher & Thurow, 1997; Marcucci Passerini et al., 1991; Musavu‐Moussavou et al., 2007) and provides additional evidence for enhanced marine productivity.
3. The subinterval above the low CaCO_3_ content (Figure 10) is marked by the sudden increase in abundance of foraminifera and return of carbonate deposition presumably after deepening of the CCD. Benthic agglutinated taxa, the epifaunal *Gavelinella* and *Stensioeina* and the opportunist calcareous *Praebulimina* show a significant increase in abundance that suggests the presence of oxic‐dysoxic bottom waters. The planktonic foraminiferal record at sites U1513 and U1516 slightly differs, pointing to differences in water column stratification. At Site U1513 the dominance of *Microhedbergella* over the other planktonic foraminiferal genera, the presence of lower mixed layer planispiral and scattered keeled taxa, and the radiolaria distributions and abundances might suggest the presence of a thick mixed layer with significant thermal differences between surface and thermocline waters. On the contrary at Site U1516 *Microhedbergella* is progressively replaced in abundance by *Muricohedbergella* to indicate a relatively stable water column with a thick mixed layer and a thin thermocline and frequent episodes of eutrophy toward the top.
  The increase in marine productivity seems more pronounced at Site U1516 and likely resulted from the delivery of continentally derived nutrients to the ocean based on the δ^13^C and δ^18^O data from foraminiferal tests (Petrizzo et al., 2021).

Relatively high terrestrial sediment influx is consistent with the proximity of the two sites to the southwestern Australian margin, with Site U1516 being the more proximal and thus more affected by episodic eutrophy as also corroborated by geochemical data and clay mineralogy proxies (Chen et al., 2022).

Although the genus *Planoheterohelix*, whose abundance indicates high productivity and low oxygen conditions, is almost absent in the lower intervals, it becomes relatively common in this interval, but it does not show the abrupt peak in abundance (*Heterohelix* shift: Leckie, 1985; Leckie et al., 1998) similar to that documented within the OAE 2 in Tethyan and Western Interior Seaway stratigraphic sections. This observation suggests that euxinia in the surface water did not develop in the Southern Hemisphere. However, because abrupt increase in abundance of biserial taxa has been also observed in Turonian intervals coinciding with normal paleoenvironmental conditions (NW Atlantic: Huber et al., 1999; Tanzania: Haynes et al., 2015; Huber et al., 2017), we believe *Planoheterohelix* was able to adapt to different water masses in a wide range of depth habitats and thus was not strictly indicative of oxygen depleted conditions.

### 5.3 Interval Above OAE 2

The termination of the OAE 2 interval (Figure 10) is identified by the carbon isotope record and its correlation with the biostratigraphic events. This level does not correspond with remarkable changes in the composition of the microfossil assemblages suggesting that the change in the δ^13^C curve reflects the global carbon cycle with minimal effects on regional conditions. That is, the paleoceanographic scenario for the interval above OAE 2 is similar to that inferred in upper subinterval of OAE 2 with a relatively stable and thermally stratified water column that was interrupted by episodic phases of enhanced eutrophy, particularly at Site U1516.

## 6 Conclusions

High resolution carbon isotope and microfossil assemblage data from Site U1513 are compared with previously published records from Site U1516. Together, these sites yield the most complete OAE 2 and Cenomanian/Turonian boundary sedimentary sections in the Southern Hemisphere and reveal a detailed history of the microfossil assemblage changes. The two sites are located 69 km apart in the Mentelle Basin in the SE Indian Ocean and were positioned at a paleolatitude of 59°–60°S during the mid‐Cretaceous.

The microfossil and carbon isotope records are compared to highlight similarities and differences between the sites and to verify the response of biota to the paleoenvironmental perturbations associated with OAE 2. The comparison between sites is based on bioevents that allow direct correlation and identification of the OAE 2 interval whose duration is estimated to be 950 ky. The onset of OAE 2 is identified according to the δ^13^C values following previous definitions and no remarkable changes are registered by the microfossils assemblages that strictly correlate with the onset and the termination of the event.

The sedimentary record corresponding to OAE 2 is marked by an interval of low CaCO_3_ content that coincides with the dominance of radiolaria and a rise in the CCD presumably resulting from ocean acidification during the thermal maximum and the δ^13^C excursion. These changes interpreted to have been forced by the high atmospheric *p*CO_2_ released by volcanic activity associated with the Central Kerguelen Plateau and other LIP eruptions. The interval of low CaCO_3_ content is more extended at Site U1513 and spans the entire interval where peak values of the δ^13^C positive excursion are registered at Site U1516 and elsewhere. Just below the interval of low CaCO_3_ content, a short hiatus of about 200 ky at Site U1513 is inferred from the age‐depth model and may result from seafloor erosion and/or winnowing and redistribution of sediments by currents.

Changes in abundance of the planktonic foraminiferal genera and the co‐occurring benthic foraminifera, radiolaria and calcispheres across OAE 2 depict a paleoceanographic scenario dominated by eutrophy resulting from a strong influence of continentally derived nutrients leading to enhanced marine productivity. The terrestrial sediment influx is consistent with the proximity of the two sites to the southwestern Australian margin and is corroborated by published geochemical and mineralogical data from Site U1516.

The distribution and abundance of the planktonic foraminiferal genera, each of them with specific paleoecological preferences, indicates that sites U1513 and U1516 were above the CCD before and during the onset of OAE 2 in a water column characterized by a predominantly eutrophic regime with variations of marine productivity and thickness of the mixed layer, as indicated by the fluctuations in abundance of the intermediate dwelling planktonic foraminifera. This phase is followed by shoaling of the CCD, as indicated by the dominance of radiolaria that reflect extremely high marine productivity and ocean acidification because of CaCO_3_ undersaturation. This extreme paleoenvironment lasted about 100 ky before returning to a relatively stable paleoceanographic regime as revealed by more diverse planktonic foraminiferal assemblages in the upper OAE 2 interval, including the occurrence of lower mixed layer to thermocline dweller taxa thriving in stratified waters. However, enhanced eutrophic episodes still occurred either because of upwelling of nutrient‐rich and δ^13^C‐depleted intermediate water masses or delivery of continental nutrients.

The sedimentation rate was higher at Site U1513 than at Site U1516 from within OAE 2 through the lowermost Turonian, indicating an increase in carbonate production and dilution by terrigenous sediments. This conclusion is corroborated by the occurrence of diverse and abundant pelagic calcifiers reflecting a more stable and stratified water column. Variations in the composition of the microfossil assemblages coupled with the calculated sedimentation rates provide support for the paleobathymetric reconstruction of the two sites, with Site U1513 located northwest of the Mentelle Basin depocenter and at a deeper depth than Site U1516. Thus, Site U1513 was probably more influenced by water circulation in the nearby Perth Abyssal Basin.

Finally, analysis of the microfossils and carbon isotope records at sites U1516 and U1513 provide a robust chronostratigraphic framework and a comprehensive understanding of the response of biota to the environmental perturbations associated with warming and high *p*CO_2_ at high latitudes in the Southern Hemisphere. The results presented here provide valuable paleontological and geochemical insights that improve our knowledge of the causes and consequences of paleoenvironmental changes associated with OAE 2 at high latitudes in comparison with concomitant records from lower latitudes.

## Data Availability

The distribution chart and absolute abundances of planktonic and benthic foraminifera, radiolaria and calcispheres, the distribution chart of calcareous nannofossils, the carbon and oxygen stable isotopes, the total organic carbon and the calcimetry data of Site U1513 are available in Petrizzo, Amaglio, et al. (2022) at PANGAEA Data Publisher for Earth & Environmental Science.
